# MHTAPred-SS: A Highly Targeted Autoencoder-Driven Deep Multi-Task Learning Framework for Accurate Protein Secondary Structure Prediction

**DOI:** 10.3390/ijms252413444

**Published:** 2024-12-15

**Authors:** Runqiu Feng, Xun Wang, Zhijun Xia, Tongyu Han, Hanyu Wang, Wenqian Yu

**Affiliations:** Qingdao Institute of Software, College of Computer Science and Technology, China University of Petroleum (East China), Qingdao 266580, China; s23070007@s.upc.edu.cn (R.F.); z23070001@s.upc.edu.cn (Z.X.); z23070070@s.upc.edu.cn (T.H.); z23070040@s.upc.edu.cn (H.W.); z23070079@s.upc.edu.cn (W.Y.)

**Keywords:** protein secondary structure prediction, multi-feature fusion, highly targeted autoencoder, deep multi-task learning, pre-trained protein language model

## Abstract

Accurate protein secondary structure prediction (PSSP) plays a crucial role in biopharmaceutics and disease diagnosis. Current prediction methods are mainly based on multiple sequence alignment (MSA) encoding and collaborative operations of diverse networks. However, existing encoding approaches lead to poor feature space utilization, and encoding quality decreases with fewer homologous proteins. Moreover, the performance of simple stacked networks is greatly limited by feature extraction capabilities and learning strategies. To this end, we propose MHTAPred-SS, a novel PSSP framework based on the fusion of six features, including the embedding feature derived from a pre-trained protein language model. First, we propose a highly targeted autoencoder (HTA) as the driver to encode sequences in a homologous protein-independent manner. Second, under the guidance of biological knowledge, we design a protein secondary structure prediction model based on the multi-task learning strategy (PSSP-MTL). Experimental results on six independent test sets show that MHTAPred-SS achieves state-of-the-art performance, with values of 88.14%, 84.89%, 78.74% and 77.15% for Q3, SOV3, Q8 and SOV8 metrics on the TEST2016 dataset, respectively. Additionally, we demonstrate that MHTAPred-SS has significant advantages in single-category and boundary secondary structure prediction, and can finely capture the distribution of secondary structure segments, thereby contributing to subsequent tasks.

## 1. Introduction

Protein is the main carrier of life activities and occupies an important position in living organisms. The structure and function of proteins are closely related, and proteins that perform similar functions usually have similar structures. Studies of protein structure are helpful in understanding the process by which proteins perform biological functions and are of great significance in genetic engineering, protein engineering, drug development and disease diagnosis. Protein structure is divided into four levels. The protein secondary structure prediction (PSSP) task is to predict the secondary structure category of each residue based on protein primary structure, i.e., the amino acid residue sequence. Secondary structure plays a connecting role in the four structural levels [1]. Accurate PSSP not only helps to understand the initial folding rules of protein sequences, but can also be used to predict the tertiary structure, interactions, domains and categories of proteins [2,3].

Each amino acid residue is represented by its single-letter abbreviation (A, C, D, E, F, G, H, I, K, L, M, N, P, Q, R, S, T, V, W, Y and X), where A to Y represent the 20 standard amino acids and X represents the non-standard amino acid. The definition secondary structure of proteins (DSSP) program [4] defines eight categories of protein secondary structure: *α*-helix (H), $3_{10}$-helix (G), *π*-helix (I), extended strand (E), *β*-bridge (B), turn (T), bend (S) and others (L). These eight categories are usually divided into three categories: H, G and I are divided into helix (H), E and B are divided into strand (E) and T, S and L are divided into coil (C). Therefore, the prediction of protein secondary structure is divided into coarse-grained three-state prediction and fine-grained eight-state prediction. Recent research shows that the accuracy limit of three-state prediction is about 92%, and the accuracy limit of eight-state prediction is 84–87% [5], which means that there is still a lot of room for improving the prediction performance of protein secondary structure.

Existing experimental methods for PSSP include NMR spectroscopy [6], X-ray diffraction [7] and circular dichroism spectroscopy [8]. Although relatively accurate results can be obtained through experimental methods, time-consuming and costly disadvantages of these methods cannot be ignored. The scale of protein sequence data is growing exponentially, and it is challenging to narrow down the information scale gap between protein sequence and structure relying solely on experimental methods. Therefore, there is an urgent need to develop efficient and accurate computational methods to predict protein secondary structure.

The encoding representation of sequences is a crucial issue in computational methods. There are two encoding features currently in common use: one is the one-hot encoding feature that contains the amino acid type information, the other is the profile feature that contains sequence evolutionary information, including position-specific scoring matrix (PSSM) and hidden Markov model (HMM) features. However, the one-hot encoding feature is sparse, and the profile feature is obtained through multiple sequence alignment (MSA) encoding, which cannot fairly represent sequences with different numbers of homologous proteins. In addition, the biomolecule information contained in the above features is so extensive that the feature space utilization of PSSP-related knowledge is poor.

In recent years, many pre-trained protein language models have been proposed, aiming to extract embedding features that can be effectively utilized by downstream tasks. Unlike profile features, the quality of embedding features is independent of protein homology. Embedding features have shown excellent results in tasks such as protein design [9] and binding site prediction [10], and have achieved the best performance in the PSSP task. In summary, it is worth developing methods that fuse embedding features to weaken the interference of the number of homologous proteins on prediction performance.

Statistical-based computational methods such as Chou–Fasman [11] and GOR [12] only analyze superficial features that have not been deeply extracted, such as statistical results of the probability that an amino acid residue belongs to each secondary structure category and protein conformation parameters, resulting in low prediction performance. At present, machine learning methods based on big data analysis have become a research hotspot of the PSSP field, among which methods based on deep neural networks have achieved state-of-the-art performance. Both the interrelationships between neighboring residues and the interrelationships between long-range residues have the potential to influence secondary structure categories. For example, helices are mainly composed of neighboring residues, whereas strands involve long-range residues. Therefore, methods based on deep neural networks mainly use the convolutional neural network (CNN) and its improvements, as well as the recurrent neural network (RNN) and its improvements, to capture local and global dependency information. However, knowledge-free guided networks [13,14] cannot balance global and local features well, and ordinary learning strategies struggle to improve the learning capability of models. This illustrates the necessity of designing knowledge-guided models and using advanced learning strategies to improve prediction performance.

For the above motivation, we propose a novel framework called MHTAPred-SS, for accurate three-state and eight-state PSSP. MHTAPred-SS is composed of a highly targeted autoencoder (HTA) and a knowledge-guided protein secondary structure prediction model based on the multi-task learning strategy (PSSP-MTL), aiming to innovate the encoding strategies of protein sequences and fully assimilate global and local features. The main contributions of this study are summarized as follows:

- We propose a novel framework, MHTAPred-SS, which effectively integrates six different feature representations to comprehensively express protein knowledge, thus improving the performance of three-state and eight-state PSSP.
- Our proposed HTA can generate highly targeted encoding features, which reduces the interference of homologous proteins and improves the feature space utilization of PSSP-related knowledge. This is a new idea for sequence encoding in the PSSP field.
- Our proposed PSSP-MTL model is based on biological knowledge to balance features of different receptive fields and exchange information between multiple tasks. To the best of our knowledge, this is the first work that introduces the multi-gate mixture-of-experts (MMoE) structure to empower secondary structure prediction with an auxiliary task.
- We demonstrate through experiments on six independent test sets that MHTAPred-SS achieves state-of-the-art performance, with significant advantages in single-category and boundary secondary structure prediction. The case study intuitively demonstrates that MHTAPred-SS can capture the distribution of secondary structure segments finely.

## 2. Related Work

Methods based on artificial intelligence algorithms can improve prediction performance while avoiding time-consuming and costly experimental processes, becoming the mainstream research direction in the PSSP field. These methods can be roughly divided into traditional machine learning methods, deep learning methods and methods based on pre-trained protein language models.

Traditional machine learning methods such as support vector machines [15,16,17], hidden Markov models [18,19] and neural networks [20,21] have been widely used in the PSSP field. These models are trained using various features. However, it is challenging to capture complex relationships between sequence features and secondary structure categories based on traditional machine learning models.

The expansion of protein databases and the improvement of computing power enable deep learning methods to be used for PSSP. For example, DNSS [22] and SPIDER2 [13] were based on deep neural networks to predict protein secondary structure. Since CNN and RNN have advantages in extracting contextual features, deep learning models based on the above two neural networks are widely used in the PSSP field. For CNN-based methods, DeepCNF [23] combined CNN with conditional neural fields and used PSSM to predict secondary structure of proteins. IGPRED [24] combined CNN with graph convolutional networks, but it did not extract global features. For RNN-based methods, SPIDER3 [25] was based on the long short-term memory (LSTM) [26] network and bidirectional recurrent neural network (BRNN) to capture global features. SPOT-1D [27] used an ensemble model of LSTM-BRNN and ResNet for protein structure prediction. Moreover, DCRNN [28], CNN_BIGRU [14] and DeepACLSTM [29] used CNN and RNN collaboratively to learn local and global features simultaneously. Multi-S3P [30] constructed three independent models based on CNN, bidirectional long short-term memory (BiLSTM) [31] and a self-attention mechanism to predict different secondary structure categories. DeepCNN [32] and MUFOLD-SS [33] were based on multi-scale convolution to extract multi-scale features. In addition, some recent methods focused on sequence feature selection, and used finalized feature subsets to predict protein secondary structure [34,35].

Pre-trained protein language models can generate embedding features that are independent of homologous proteins. Many methods based on pre-trained protein language models have been proposed to achieve the closest possible prediction effect for different sequences. DML_SS [36] used the pre-trained protein language model ProtT5-XL-U50 [37] to generate embedding features, and used a deep metric learning prediction model to avoid the time-consuming search process of the *k*-nearest neighbor algorithm. AttSec [38] also used ProtT5-XL-U 50 and obtained attention maps from transformer encoder to predict protein secondary structure. SPOT-1D-LM [39] combined one-hot encoding features with embedding features extracted from two pre-trained protein language models (ProtTrans [37] and ESM-1b), and was able to achieve performance comparable to profile feature-based methods. In addition, LIFT_SS [40] focused on fine-tuning pre-trained protein language models, and used different fine-tuning methods for the PSSP task.

In short, compared with traditional machine learning methods, deep learning methods can efficiently process high-dimensional data and have more powerful modeling and expression capabilities. Prediction methods based on pre-trained protein language models have achieved state-of-the-art performance, but they mainly use embedding features without fusing features such as profile features and physicochemical properties. Considering the advantages of multi-feature fusion in comprehensively expressing protein knowledge, we fused six different features and propose HTA to improve feature space utilization. In addition, we designed a network architecture under the guidance of biological knowledge and used the multi-task learning (MTL) strategy to improve prediction performance. Compared with simple stacked network architectures and common learning strategies, our method enhances the ability to extract complex features such as local and global information of protein sequences and improves the learning ability of the model.

## 3. Results and Discussion

### 3.1. Data Analysis

#### 3.1.1. Protein Secondary Structure Segment Length Pattern

**Figure 1:**
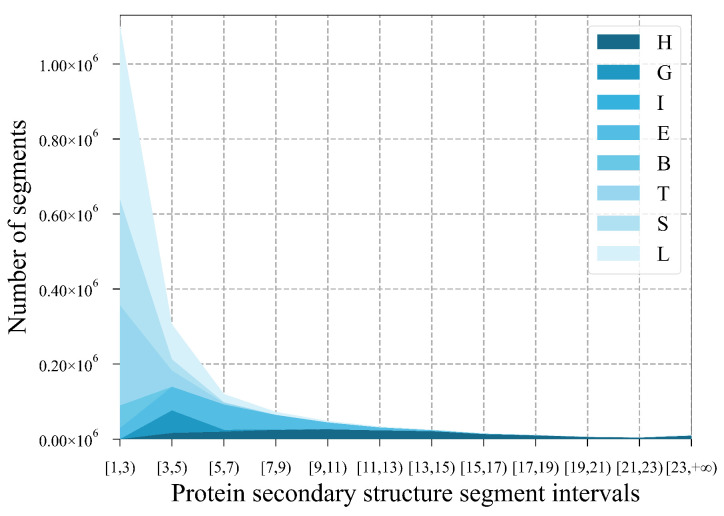
The secondary structure segment length pattern. The horizontal axis represents the interval to which the secondary structure segment length belongs, and the vertical axis represents the number of secondary structure segments.

As shown in Figure 1, lengths of secondary structure segments in the used datasets were mostly between 1 and 11. We selected scales from {1, 3, 5, 7, 9, 11} amino acid residues for multi-scale convolution to extract multi-scale local features of sequences. Since a few secondary structure segments were relatively long and different segments may interact with each other, we designed the combined time series module to balance global and multi-scale local features.

#### 3.1.2. Correlation Between Secondary Structure and Relative Solvent Accessibility (RSA)

Access surface area (ASA) is the surface area of residues in contact with solvents. The RSA of each residue is defined as the ratio of its ASA to the largest ASA found for this kind of residue. We analyzed the correlation between secondary structure and RSA as the knowledge guidance for the application of the MTL strategy.

**(1)**  **Visualization method**

**Figure 2:**
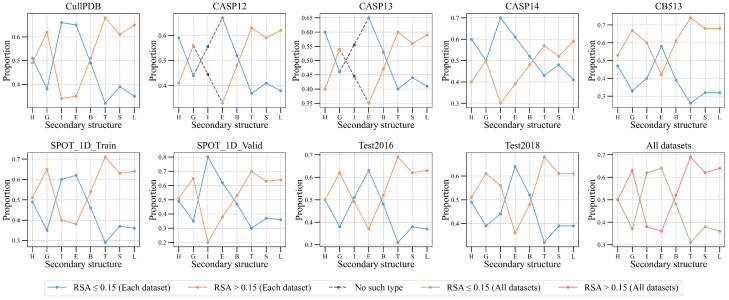
The correlation between secondary structure and RSA. Each horizontal axis represents the eight secondary structure categories, and each vertical axis represents the proportion of RSA ≤ 0.15 and RSA > 0.15. The gray dashed lines indicate that there are no amino acid residues belonging to this secondary structure category in the dataset.

As shown in Figure 2, the proportions of RSA ≤ 0.15 and RSA > 0.15 among residues in the same secondary structure category are roughly the same. As the secondary structure category changes, trends of the proportions are also roughly the same. We consider that there is a correlation between secondary structure and RSA.

**(2)**  **Statistical analysis method**

Cramer’s V correlation coefficient can evaluate the correlation between discrete variables. The contingency table of secondary structure and RSA is shown in Table 1. The process of calculating Cramer’s V correlation coefficient based on the contingency table is as follows:

(1) $$E_{ij}=\frac{{sum}_{i}\times {sum}_{j}}{sum}$$

(2) $$Cramer^{\prime }s\;V=\sqrt{\frac{\sum_{i=1}^{m}\sum_{j=1}^{n}\frac{{\left(O_{ij}-E_{ij}\right)}^{2}}{E_{ij}}}{sum\times \left(\min\left(m-1,n-1\right)\right)}}$$

where $sum_{i}$ is the sum of data in the *i*-th row, $sum_{j}$ is the sum of data in the *j*-th column, sum is the sum of all data, $E_{ij}$ is the expected value of the data in the *i*-th row and *j*-th column, $O_{ij}$ is the true value of the data in the *i*-th row and *j*-th column, *m* is the number of rows and *n* is the number of columns (the above calculations do not include the Sum row and Sum column).

We calculate that the Cramer’s V correlation coefficient between secondary structure and RSA is 0.2264; it can be considered that there is a strong but not very strong correlation between the two discrete variables [41], so it is suitable to implement the soft sharing of bottom parameters based on the MMoE structure. A detailed interpretation of Cramer’s V correlation coefficient is provided in Supplementary Table S1.

#### 3.1.3. Boundary Amino Acid Residues

We defined amino acid residues that satisfy the following three types of situations as boundary amino acid residues:

***Type 1:*** at the beginning or end of protein sequences.

***Type 2:*** the secondary structure categories of the two residues before and after are different.

***Type 3:*** the secondary structure categories of the two residues before and after are the same, but its own secondary structure is different from the secondary structure of the residue before it.

**Figure 3:**
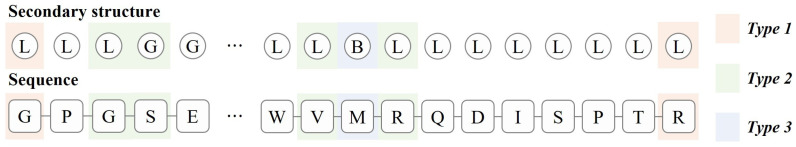
Examples of boundary amino acid residues.

Examples of boundary amino acid residues are shown in Figure 3, and the number of boundary amino acid residues contained in six independent test sets is shown in Table 2. Contextual information is used to infer secondary structure categories, and boundary amino acid residues are at critical points in protein sequences. Therefore, it is extremely challenging to predict secondary structure categories of boundary amino acid residues.

### 3.2. Ablation Study

An ablation study aims to explore the impact of removing or changing a component on the performance of the model based on the control variable method. In this work, changes in hyperparameters, critical components, feature combinations and learning strategies we explored will cause changes in a certain model component (neuron, network layer, component module, sequence feature, etc.) while controlling other variables. Therefore, we classified experiments that explore the impact of the above variables on model performance as the ablation study.

We performed extensive experiments on two sets of validation sets and test sets (CB513 [42] and TEST2016 [27]). In this paper, the first validation set and test set are denoted as Validation set1 and Test set1, respectively, and the second validation set and test set are denoted as Validation set2 and Test set2, respectively. To explore a wider range of multi-scale combinations, all experiments before the different residual convolution scales experiment were based on four scales: 3, 5, 7 and 9. For each experiment, we performed it three times independently using the same random seed (42) so that the parameters of the model were initialized the same each time, thus achieving a fair comparison. We used the early stopping mechanism with the patience parameter set to 3, which can effectively prevent the overfitting problem of the model and ensured that the model of each experiment achieved convergence. We reported the average prediction performance among three experimental results, and the reported results were statistically reliable.

#### 3.2.1. Impact of Model Hyperparameters

**The number of filters in the residual dynamic one-dimensional convolution (DyConv1D) block** (*B*)**.** We set *B* in the range of {1, 2, 3, 4}, and the experimental results are shown in Table 3. When B=3, the model achieves the best prediction performance on most datasets. However, when B=4, the prediction performance decreases. This may be because, when the number of filters is too small, it is difficult to express specific information for sequences, while too many filters will introduce redundant parameters that are difficult to optimize with limited training data and learning time.

**Figure 4:**
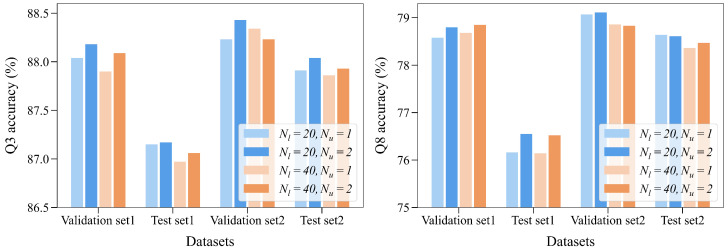
The prediction performance of models under BiLSTM with different numbers of layers and hidden units.

**The number of BiLSTM layers** ($N_{l}$) **and hidden units** ($N_{u}$)**.** We selected $N_{l}$ from {20, 40} and $N_{u}$ from {1, 2}. As shown in Figure 4, the model achieves the best performance when $N_{l}=20$ and $N_{u}=2$. The reason may be that too few parameters make the model learn global features difficultly, while too many parameters make the model overfit.

**Different residual convolution scales.** Given the knowledge of secondary structure segment length pattern (Section 3.1.1), we built single-scale residual convolution modules based on the six scales of 1, 3, 5, 7, 9 and 11 to replace the multi-scale residual convolution module. The experimental results are shown in Table 4. The experimental results of the three scales 1, 5 and 9 almost cover the best prediction performance of single-scale models on all datasets, so we used these three scales to build the multi-scale residual convolution module. As can be seen from Table 4, the model achieves the best prediction performance on most datasets when using the multi-scale residual convolution module.

**Figure 5:**
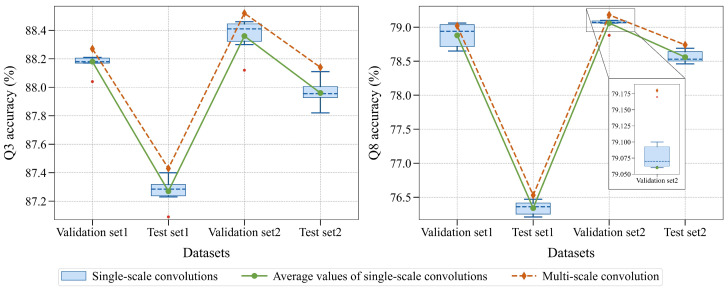
The prediction performance of models when using different residual convolution scales. The expanded chart is the eight-state prediction experimental results based on models of different scales on Validation set2.

As shown in Figure 5, the prediction performance of the model based on the multi-scale residual convolution is better than the average prediction performance of single-scale models. The reason may be the design of the multi-scale residual convolution module is guided by the knowledge of secondary structure segment length pattern, so that it can learn local features at multiple pivotal receptive fields.

**The number of expert networks** (*E*)**.** We vary *E* in the range of {1, 2, 3, 4}, and the experimental results are shown in Table 5. When E=3, the model achieves the best prediction performance on most datasets. The reason may be that the number of tasks we involve is small, and three expert networks are enough to implement the information communication between tasks.

**Figure 6:**
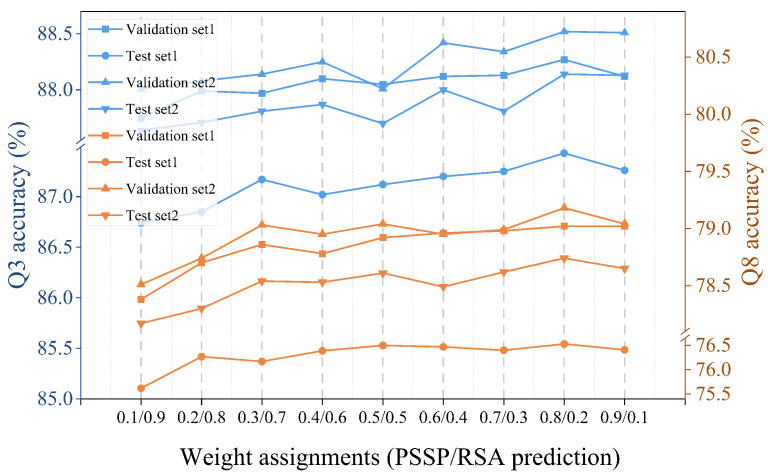
The prediction performance of models under different weight assignments.

**Different weights for losses.** For MTL models, it is necessary to fuse losses of different tasks. Fusion methods that assign weights to task losses are widely used. Considering that we had a small number of tasks, we adjusted weights manually with low cost. As shown in Figure 6, the prediction performance changes slowly under different weight assignments. When the loss weight of the PSSP task is 0.8, the model achieves the best prediction performance.

#### 3.2.2. Impact of Critical Components

To demonstrate the necessity of critical components in the PSSP-MTL model and explore their contribution to prediction performance, we performed experiments using six variants of the PSSP-MTL model.

The experimental results of disabling each component and using only one component are shown in Table 6 and Table 7, respectively. As can be seen from Table 6, when disabling the combined time series module or the parameter sharing module, the prediction performance decreases significantly, about 0.34%, while when disabling residual connections, the decrease is only about 0.05%. It can also be seen from Table 7 that the combined time series module and parameter sharing module contribute significantly to the model prediction performance. The reason may be that the secondary structure category of each amino acid residue is closely related to the global features of sequences, and the combined time series module can effectively extract global features of protein sequences and achieve more accurate secondary structure identification. In addition, the parameter sharing module implements parameter soft sharing between tasks, which can not only retain the specificity of the features required for each task, but also enrich its feature representation through information exchange between tasks. Although the prediction performance of the model is relatively low when using only the multi-scale residual convolution module, it can also improve the prediction performance when used in conjunction with other components. This proves that the multi-scale local features extracted by the multi-scale residual convolution module can be effectively applied to the subsequent parameter sharing and global feature extraction process.

The PSSP-MTL model consistently outperforms its variant models, which demonstrates the effectiveness and necessity of the critical components. These components enable the PSSP-MTL model to balance global and multi-scale local features, achieve information exchange between tasks and be stably trained.

#### 3.2.3. Impact of Feature Combinations

We explored the prediction performance under different input feature combinations of the PSSP-MTL model, and the experimental results are shown in Table 8. As can be seen from the table, the embedding feature with a length of 1280 contributes the most to the improvement of prediction performance, which benefits from the rich information learned from massive data. When the highly targeted encoding feature or the profile feature is disabled, prediction performance decreases by a similar amount. However when physicochemical properties of length 7 are disabled, prediction performance decreases slightly, and even the Q3 accuracy on Validation set2 has a small improvement of 0.09%. Although the length of the highly targeted encoding feature is only 4, it can play a comparable role with profile features. This indicates that the highly targeted encoding feature has extremely high feature space utilization of PSSP-related knowledge.

Since the lengths of both the highly targeted encoding feature and the physicochemical properties are very short, using only these two features will result in low prediction performance. However, when these two features are combined with other features, the prediction performance of the model can be significantly improved, especially the highly targeted encoding feature. This demonstrates that there is a strong complementarity between the highly targeted encoding feature and other features, which is of great significance in the PSSP field. It can be seen from Table 8 that the PSSP-MTL model achieves the best prediction performance when all features are enabled. Therefore, it can be proved that multi-feature fusion can comprehensively reflect protein knowledge.

#### 3.2.4. Impact of Learning Strategies

**Figure 7:**
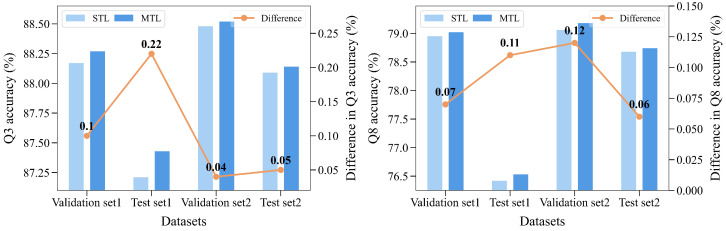
The prediction performance under different learning strategies. “Difference” represents the difference between the prediction performance of the MTL model and the STL model.

To demonstrate the superiority of the MTL strategy, we performed experiments using different learning strategies. As shown in Figure 7, the MTL strategy always outperforms the single-task learning (STL) strategy, and the difference between two strategies ranges from 0.04% to 0.22%. This demonstrates that the MTL strategy can effectively implement the communication between tasks, enabling the auxiliary task to assist in the PSSP task.

### 3.3. Comparison with Existing Prediction Methods for PSSP

In this section, we compared MHTAPred-SS with 10 state-of-the-art methods on six independent test sets. Experiments on single-category and boundary secondary structure prediction were performed on TEST2016, which contains the largest number of sequences, general residues and boundary residues.

Among 10 baselines, DeepCNN [32] used multi-scale convolution to extract features at different receptive fields. MUFOLD-SS [33] was a predictor based on the inception-inside-inception architecture. SPIDER3 [25] and SPOT-1D [27] combined LSTM and BRNN to extract bidirectional global features. DCRNN [28], CNN_BIGRU [14], DeepACLSTM [29] and Multi-S3P [30] effectively alleviated limitations of single models with the collaborative operation of CNN and RNN. In addition, DML_SS [36] and LIFT_SS [40] used pre-trained protein language models to generate embedding features and improved prediction performance based on dense vectors of residues. We represent DML_SS based on embedding features as DML_SS^embed^ and DML_SS based on ensemble models as DML_SS^ensemble^.

To make fair comparisons with existing models, the PSSP-MTL model used the same two sets of datasets as DML_SS [36]. For convenience, we refer to these two sets of datasets as DS1 and DS2. We show the experimental result based on the same dataset. These baselines were restricted in terms of training platforms, computing resources and input feature factors. For the six methods, DCRNN, DeepCNN, CNN_BIGRU, MUFOLD-SS, DeepACLSTM and DML_SS^embed^, we report their prediction performance on test sets of DS1 and DS2, while, for other predictors based on complex factors, we report their prediction performance on test sets of DS2 (TEST2016 and TEST2018).

#### 3.3.1. Comparison of Overall Prediction Performance

Table 9 and Table 10 report the three-state and eight-state prediction performance of MHTAPred-SS and 10 baselines on six independent test sets. MHTAPred-SS significantly outperforms the baselines in both three-state and eight-state prediction, achieving state-of-the-art performance of the PSSP task. It can be observed that MHTAPred-SS shows a significant improvement in more critical segment overlap (SOV) metrics. Therefore, prediction results of MHTAPred-SS can more fully reflect the distribution of secondary structure segments, and can play an important supporting role in subsequent tasks. It can be seen from Table 10 that MHTAPred-SS outperforms DML_SS^ensemble^, which also reflects the powerful competitiveness of MHTAPred-SS.

#### 3.3.2. Comparison of Single-Category Prediction Performance

Considering that both DML_SS^embed^ and MUFOLD-SS extract multi-scale features and have relatively outstanding prediction performance on six independent test sets, comparative experiments were performed with these two methods after this section.

**Figure 8:**
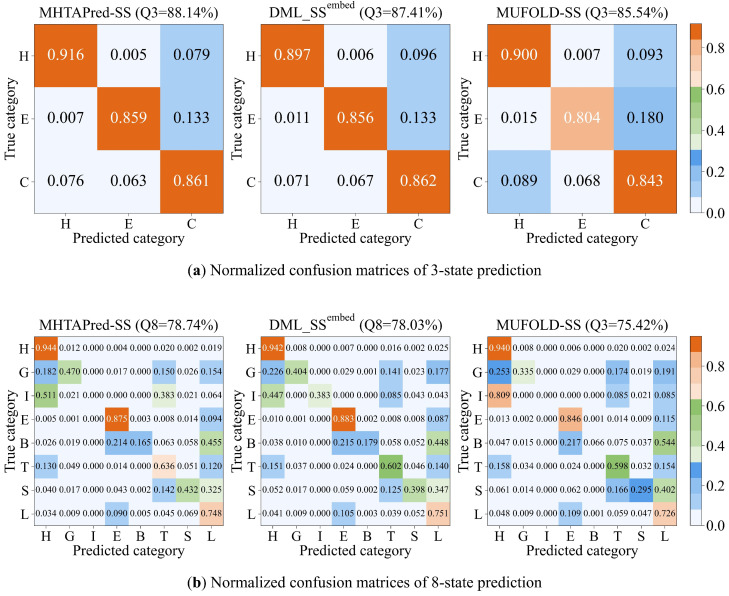
Normalized confusion matrices of three-state (**a**) and eight-state (**b**) prediction on TEST2016.

The normalized confusion matrices of three-state and eight-state prediction are shown in Figure 8a and Figure 8b, respectively. MHTAPred-SS achieves the best prediction performance for most secondary structure categories and DML_SS^embed^ outperforms MUFOLD-SS. However, there are some error-prone secondary structure categories in prediction results: G is mainly incorrectly predicted as H, T and L; I is mainly incorrectly predicted as H; and B and S are mainly incorrectly predicted as L.

We calculate based on the dataset used that there are 2,000,534, 1,344,562, 1,279,462, 644,956, 473,755, 224,006, 61,217 and 1034 amino acid residues belonging to H, L, E, T, S, G, B and I categories, respectively. It can be seen that the number of amino acid residues belonging to the S, G, B and I categories is relatively small, while the number of other categories is about seven times that of them. Therefore, the reason why these four categories are easily mispredicted may be due to the fact that there are too few residues belonging to these categories. We believe that a potential solution is to adopt advanced data augmentation methods to balance the number of occurrences of each secondary structure category. It is also possible to design a loss function for the unbalanced dataset in the PSSP task, emphasizing the loss caused by secondary structure labels with few occurrences, thereby strengthening the learning ability of the model for these categories. In future work, we will optimize our method for these error-prone secondary structure categories, aiming to further balance and improve the prediction accuracy of different secondary structure categories while maintaining or improving the overall prediction performance.

#### 3.3.3. Comparison of Boundary Prediction Performance

The boundary prediction and overall prediction accuracy on TEST2016 is shown in Table 11. Boundary amino acid residues are at critical points of protein sequences, and it is extremely difficult to predict their secondary structure categories (Section 3.1.3). Although the boundary prediction accuracy of each model is lower than the corresponding overall prediction accuracy, MHTAPred-SS still achieves the best boundary prediction performance. The boundary prediction accuracy of MHTAPred-SS achieves an improvement of about 4.3% compared to MUFOLD-SS, and an improvement of about 1% compared to DML_SS^embed^. This demonstrates that MHTAPred-SS can capture more refined features and has significant advantages in boundary secondary structure prediction. The advantages of MHTAPred-SS in predicting boundary secondary structures make its prediction results of great significance for identifying potential active sites and sensing the interactions between drug molecules and targets.

#### 3.3.4. Case Study

**Figure 9:**
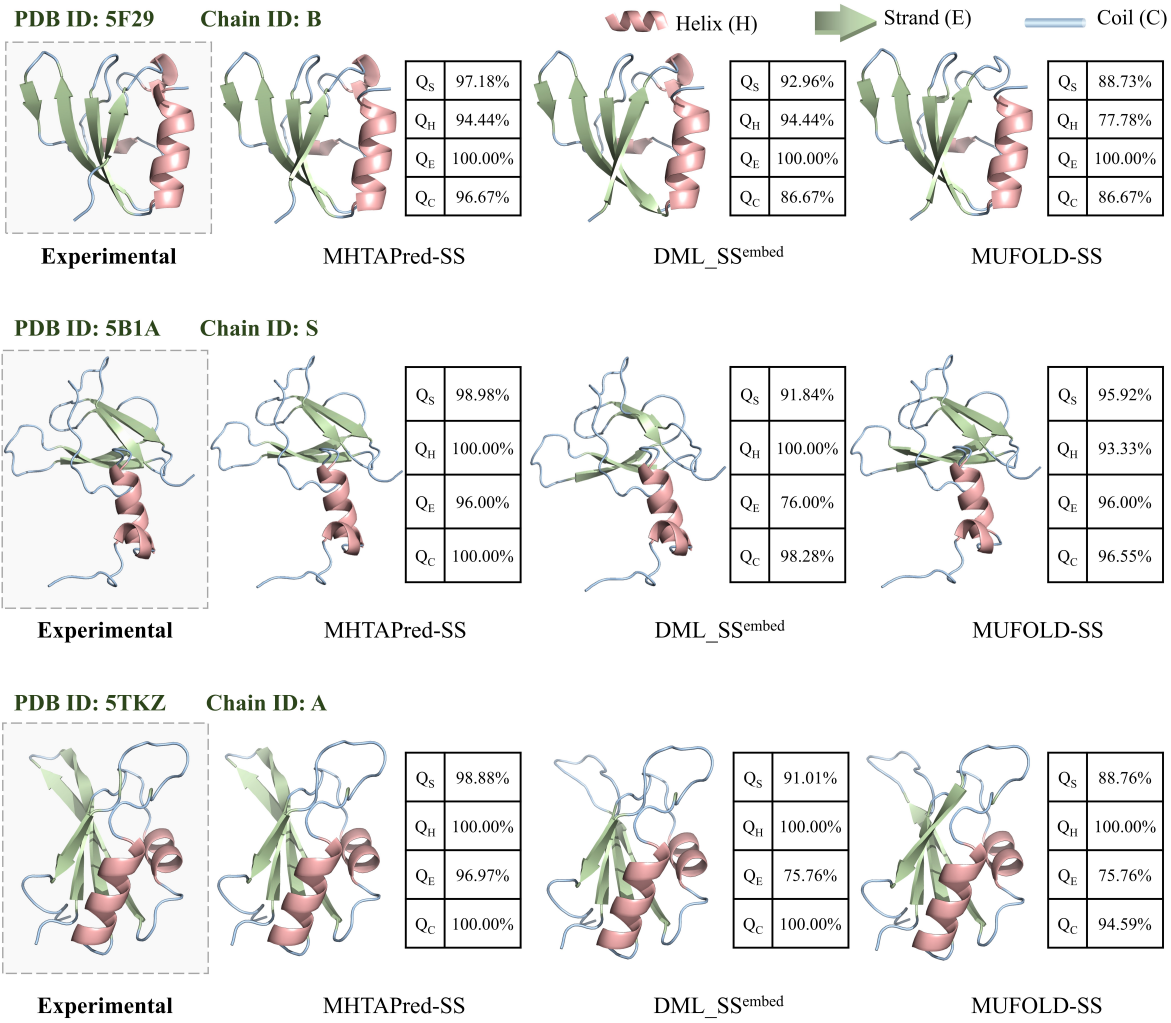
Visualization of secondary structure prediction results from different methods. The dashed box shows the biological experimental results.

As shown in Figure 9, we select three representative protein sequences belonging to different categories and use PyMol [43] to visualize prediction results of different methods, where $Q_{S}$ represents the accuracy of secondary structure prediction on a single sequence, and $Q_{H}$, $Q_{E}$ and $Q_{C}$ represent the prediction accuracy of helix, strand and coil secondary structures on a single sequence, respectively.

The first sequence we selected is the “B” chain of the protein with PDB ID “5F29”, its expression system is E. coli O103. The second sequence we selected is the “S” chain of the protein with PDB ID “5B1A”, which is present in Bos taurus. Moreover, the third sequence we selected is the “A” chain of the protein with PDB ID “5TKZ”, which is expressed in E. coli BL21 (DE3). The above three proteins belong to a transport protein, oxidoreductase and splicing protein, respectively.

It can be seen from Figure 9 that prediction results of MHTAPred-SS are almost the same as experimental results, and it can maintain extremely high stability under different states. The prediction accuracy of MHTAPred-SS on the three sequences achieves 97.18%, 98.98% and 98.88%, respectively, and MHTAPred-SS outperforms other models in the prediction of helix, strand and coil categories. In particular, for the coil category of the “5F29” protein and the strand category of the “5TKZ” protein, the prediction accuracy of our MHTAPred-SS is 10% and 21.21% higher than that of other models, respectively, and for the helix category of the three sequences, it also achieves a fairly high prediction accuracy. It can be observed that MHTAPred-SS can comprehensively improve the prediction performance of each secondary structure category and have better generalization performance for different protein samples. However, DML_SS^embed^ and MUFOLD-SS tend to confuse strand and coil, and MUFOLD-SS is insensitive to interactions of neighboring residues, thus incorrectly predicting helix as coil. In summary, MHTAPred-SS can flexibly and accurately perceive the distribution of secondary structure segments, and its prediction results can be used as the basis for determining the overall protein structure.

Since the secondary structure prediction of boundary amino acid residues is challenging, we defined samples in which boundary amino acid residues account for a relatively high proportion in the full sequence as “difficult proteins” and explored the prediction performance of different models in this case. TEST2016 contains a total of 1213 protein sequences, and the average proportion of boundary amino acid residues of a sequence is about 31.99%. We re-divided TEST2016 and classified the samples with a proportion of boundary amino acid residues greater than the average as “difficult proteins”, which contains a total of 664 protein sequences. For the three-state prediction of difficult protein samples, the prediction accuracy values of MHTAPred-SS, DML_SS^embed^ and MUFOLD-SS are 87.33%, 86.67% and 84.35%, respectively. For the eight-state prediction of difficult protein samples, the prediction accuracy values of MHTAPred-SS, DML_SS^embed^ and MUFOLD-SS are 77.90%, 77.32% and 74.24%, respectively. Since difficult protein samples contain more mutation points, the prediction accuracy for them will be lower than the prediction accuracy for all samples. However, MHTAPred-SS is still better than other models on difficult protein samples.

**Figure 10:**
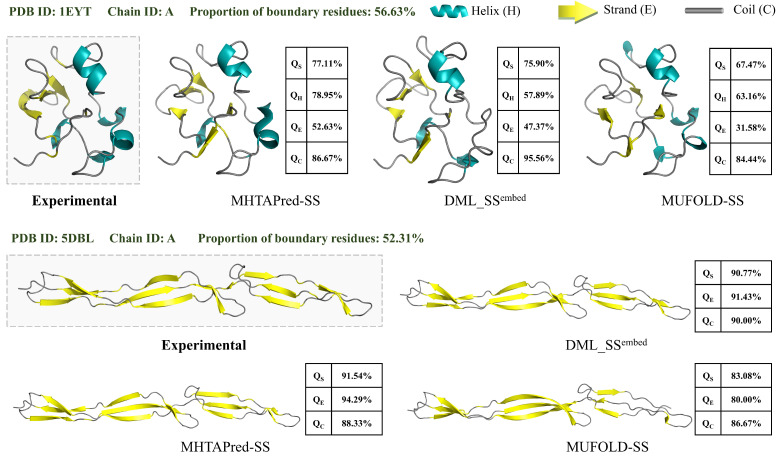
Visualization of secondary structure prediction results for difficult proteins. The dashed box shows the biological experimental results.

In addition, we selected two difficult proteins in which the proportion of boundary amino acid residues is more than 50% and visualized their prediction results. As shown in Figure 10, the first sequence is the “A” chain of the protein with PDB ID “1EYT”, and its boundary amino acid account for 56.63%. The second sequence is the “A” chain of the protein with PDB ID “5DBL”, the proportion of boundary amino acids is 52.31%. It can be seen from the figure that, for difficult proteins with such a large proportion of boundary amino acid residues, MHTAPred-SS can flexibly predict the mutation points of the secondary structure, and it still outperforms other models. In terms of the prediction of these two difficult proteins, DML_SS^embed^ tends to mispredict other secondary structure categories as coil, resulting in higher $Q_{C}$ and lower $Q_{H}$ and $Q_{E}$, and MUFOLD-SS has lower prediction performance for all secondary structure category. In summary, MHTAPred-SS has strong robustness and can relatively accurately locate the change sites of protein secondary structure, thus improving the prediction performance for difficult proteins.

### 3.4. Comparison with Colab Version of AlphaFold2

AlphaFold2 [44] can determine the overall protein structure and provide accurate protein structure prediction results based on a large amount of biological data, and it does not belong to the PSSP method in a strict sense. It requires a huge amount of training resources and data. The complete dataset is about 2.62 TB in size. It requires 85 GB of RAM and A100GPU to run its original program on Google Cloud, and the generated model parameters are about 5.3 GB in size. However, our MHTAPred-SS does not require a massive dataset during operation, and, under the configuration described in Section 4.5, it requires at most 60 GB of RAM and 13 GB of graphics memory, and the generated model parameters are about 331 MB in size. Therefore, MHTAPred-SS has the advantages of being lightweight and low in hardware resource consumption; it can be applied to a wider range of computing environments.

Given the limitations of storage and computing resources, we reproduced the Colab version of AlphaFold2 locally (LocalColabFold) [45] (https://github.com/YoshitakaMo/localcolabfold (accessed on 7 December 2024)), which is an efficient encapsulation of AlphaFold2. For a fair comparison, we filtered out sequences from CASP12, CASP13, CASP14, CB513, TEST2016 and TEST2018 that could not be predicted by LocalColabFold due to the presence of a large number of non-standard amino acid residues, and used the remaining 2092 samples for testing. We used the DSSP program [4] to extract protein secondary structure information based on the protein tertiary structure model predicted by LocalColabFold, and then compared it with our MHTAPred-SS. On these 2092 samples, the three-state and eight-state prediction accuracies of LocalColabFold were 94.31% and 89.46% respectively, and the three-state and eight-state prediction accuracies of MHTAPred-SS were 87.77% and 77.96% respectively. It can be seen that LocalColabFold can achieve higher prediction performance thanks to the MSA algorithm based on a large-scale database, massive training data, and advanced storage and computing resources. However, our MHTAPred-SS can significantly improve the prediction speed, and, in terms of predicting certain orphan proteins, MHTAPred-SS had better prediction results.

We randomly selected five sequences of length 50, 100, 150 and 200 from 2092 samples and tested the prediction time of MHTAPred-SS and LocalColabFold. We performed ten independent repeated experiments on each sequence, and the average prediction time for each sequence of a specific length is shown in Table 12. It can be seen that, whether it is a three-state prediction or eight-state prediction, our MHTAPred-SS can obtain secondary structure information in a very short time, which is about 233 times faster than LocalColabFold. In addition, the average growth rate of LocalColabFold’s prediction time is about 22.551%, which is about two times that of MHTAPred-SS. This indicates that, as the sequence length grows, the prediction speed of LocalColabFold will slow down significantly and it is difficult to obtain secondary structure information in real time.

**Figure 11:**
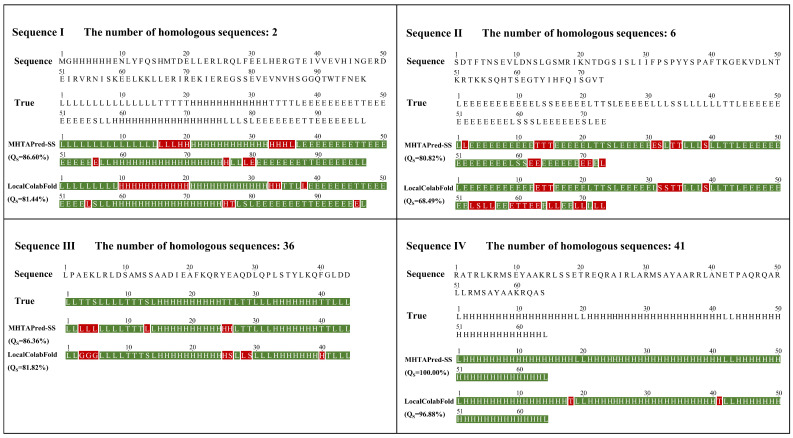
Prediction results for orphan proteins. The green segments indicate the correct predictions, and the red segments indicate the wrong predictions.

LocalColabFold finds an average of 5822 homologous proteins for each sample. The MSA results between the target protein and homologous proteins can help model the overall structure of the target protein. However, for some orphan proteins, LocalColabFold can only find a small number of homologous proteins, so the information contained in their MSA results is limited. We selected four orphan proteins, and LocalColabFold found two, six, thirty-six and forty-one homologous proteins for them, respectively. The prediction results for the orphan proteins are shown in Figure 11. As can be seen from the figure, for these orphan proteins, the prediction performance of MHTAPred-SS is less affected by the number of homologous proteins, and it can more accurately identify the secondary structure category of each amino acid residue, thereby improving the prediction performance.

## 4. Materials and Methods

### 4.1. Datasets

The PSSP-MTL model uses the same two sets of datasets as DML_SS (D1 and D2). The detailed description of independent test sets is shown in Table 13. CASP12-14 is from the critical assessment of structure prediction (CASP) competition, and sequences with more than 25% homology in each test set were deleted. CB513 was built by Cuff and Barton and has been widely used in the PSSP field [42]. The training and validation sets of DS1 were downloaded by the PISCES server [46] under conditions that homology < 25%, resolution < 2.5 Å and R-value < 1.0. We removed sequences that did not contain secondary structure information or were shorter than 50 or longer than 800 from the dataset, resulting in 12510 protein sequences, denoted as CullPDB in this paper. In CullPDB, sequences that were more than 25% homologous to each test set were deleted. Finally, sets containing 12376, 12453, 12504 and 12012 sequences were left for CASP12, CASP13, CASP14 and CB513, respectively. We randomly selected 512 sequences from the intersection of the above four sets as the validation set, and remaining sequences in each set were used as the training set. DS2 contained two test sets, TEST2016 and TEST2018, and we used a default training set containing 10029 sequences and validation set containing 983 sequences of these two test sets [27], represented as SPOT_1D_Train and SPOT_1D_Valid, respectively.

In the first set of datasets for HTA, we used the intersection of four training sets in DS1 as the training set, and set the same validation and test sets as DS1. The second set of datasets for HTA was DS2.

### 4.2. Feature Representation

To comprehensively express protein knowledge, we used six sequence features: PSSM profile features, HMM profile features, physicochemical properties, embedding features, one-hot features and highly targeted encoding features. Assuming that the length of a protein sequence was *L*, then the sizes of its PSSM and HMM feature were L×20 and L×30, respectively. Each PSSM feature was generated using the PSI-BLAST program [47] through two iterative searching against the Uniref50 database with an E-value of 0.001. Each HMM feature was generated through four iterations using the HHblits v3.3.0 program [48] based on default parameters of the uniprot20_2013_03 database. It should be noted that the sequences with fewer homologous proteins had lower-quality profile features.

We used four homologous protein-independent features: physicochemical properties, embedding features, one-hot features and highly targeted encoding features. Among them, physicochemical properties included sheet probability, helix probability, isoelectric point, hydrophobicity, van der Waals volume, polarizability and graph shape index, and we used ESM-2 [49] to generate the embedding feature of size L×1280. In addition, the one-hot feature with size L×21 was the input of HTA, and the highly targeted encoding feature with size L×4 was the output of HTA encoder.

### 4.3. The Workflow of MHTAPred-SS

**Figure 12:**
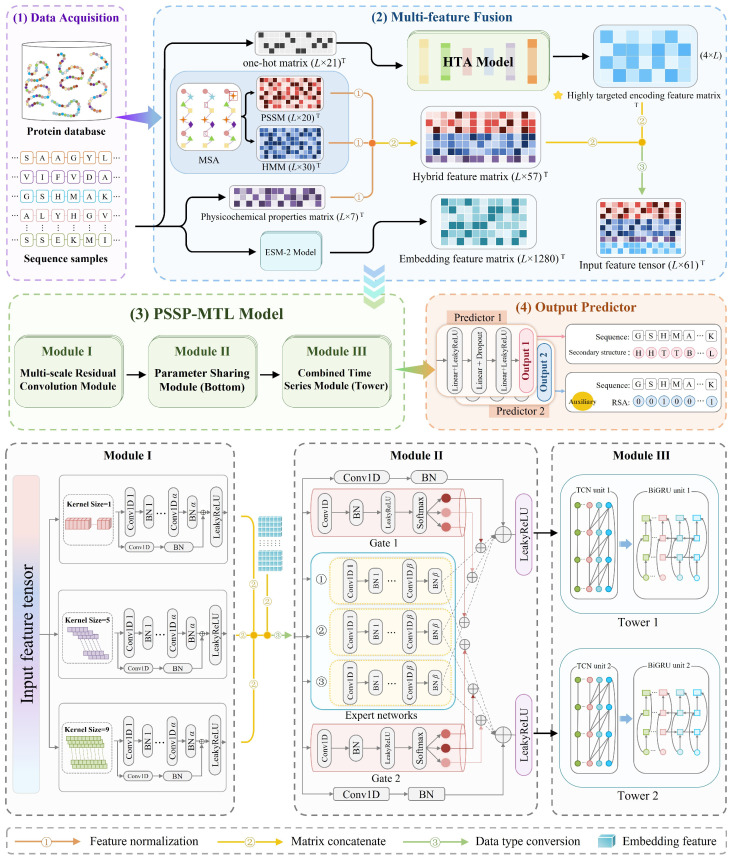
The workflow of MHTAPred-SS. Our proposed MHTAPred-SS consists of four key components: (**1**) data acquisition: two sets of datasets are obtained for model training, validation and testing; (**2**) multi-feature fusion: six different features are obtained using five methods; (**3**) PSSP-MTL model: the PSSP-MTL model consists of three modules and (**4**) output predictor: the output predictor simultaneously outputs predicted results of secondary structure and RSA.

The workflow of MHTAPred-SS proposed in this paper is shown in Figure 12, which comprises four key components: (1) data acquisition, (2) multi-feature fusion, (3) PSSP-MTL model and (4) output predictor. The detailed description of MHTAPred-SS is as follows.

#### 4.3.1. HTA Model

**Figure 13:**
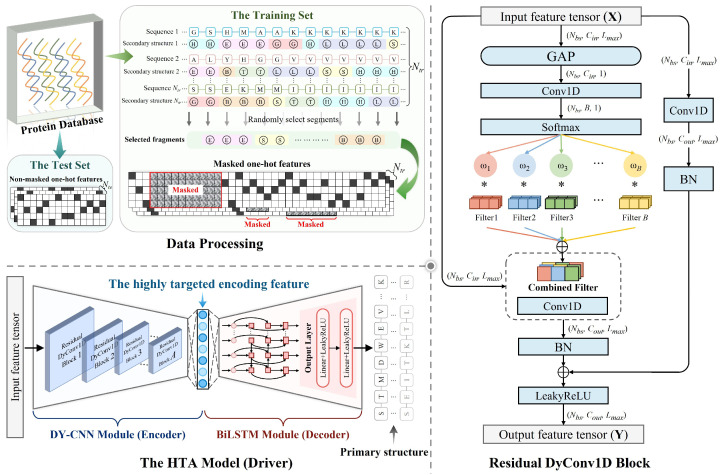
The operating mechanism of HTA. HTA is divided into two parts: encoder (DY-CNN module) and decoder (BiLSTM module), which reconstruct the primary structure information of each protein.

We designed HTA as a driver to map one-hot features into low-dimensional highly targeted encoding features. In the HTA model, the dynamic convolutional neural network (DY-CNN) module serves as the encoder and the BiLSTM module serves as the decoder. The operating mechanism of HTA is shown in Figure 13. The training set and test set used by HTA contained $N_{tr}$ and $N_{te}$ protein sequences, respectively. In the training stage, we randomly selected a residue segment with a single secondary structure category for each sequence to mask. Since the testing stage is only for obtaining the output features of the encoder, using non-masked data can avoid ignoring partial residues, making obtained encoding features more comprehensive.

Different from conventional general-purpose autoencoders, our HTA is a PSSP task-specific autoencoder. It is based on the mask operations on secondary structure segments and the flexible parameter allocation of dynamic convolution, and is highly targeted to both protein secondary structure information and sample information. In addition, HTA does not require the complex MSA process. It accepts a single sequence as input and generates highly targeted encoding features that are independent of homologous proteins, that is, it can fairly encode each protein sequence.

**(1)**  **DY-CNN module (Encoder)**

The DY-CNN module is built based on the dynamic convolution [50]. Different from traditional convolution, it sets an attention mechanism on the sample dimension, which can assign specific filter parameters to each sample.

The DY-CNN module we designed contains *A* residual DyConv1D blocks. In each residual DyConv1D block, the global average pooling results of the input features $X\in R^{N_{bs}\times C_{in}\times L_{max}}$ are processed by a one-dimensional convolutional layer (Conv1D) and a Softmax operation to obtain weights corresponding to *B* filters. Then, Combined Filter can be generated by weighted addition of *B* filters. It should be noted that $N_{bs}$ is the batch size, $C_{in}$ is the number of input feature channels and $L_{max}$ is the maximum sequence length in each batch. The input features are convolved using the Combined Filter, and the features obtained through the batch normalization layer (BN) are added element by element to the features transmitted by the residual connection. The LeakyReLU function is used for nonlinear mapping to obtain the output features $F_{Output}\in R^{N_{bs}\times C_{out}\times L_{max}}$, i.e., the highly targeted encoding features, where $C_{out}$ is the number of output feature channels. Particularly, we set a small $C_{out}$ to improve the feature space utilization of PSSP-related knowledge, which is formulated as follows:

(3) $${MAT}_{\omega }=Softmax\left(Conv\left(GAP\left(X\right)\right)\right)$$

(4) $${MAT}_{\omega }=\left[\left[\omega_{1}\right],\left[\omega_{2}\right],\cdots \left[\omega_{B}\right]\right]$$

(5) $${MAT}_{FLT}=\left[\left[{FLT}_{n}^{1}\right],\left[{FLT}_{n}^{2}\right],\cdots \left[{FLT}_{n}^{B}\right]\right]$$

(6) $$Comb\_FLT={MAT}_{\omega }{MAT}_{FLT}^{T}$$

(7) $$F_{Output}=LeakyReLU\left(BN\left({Conv}_{Comb\_FLT}\left(X\right)\right)⊕BN\left(Conv\left(X\right)\right)\right)$$

where GAP is the global average pooling operation, Conv is the one-dimensional convolution operation, BN is the batch normalization operation, ${MAT}_{\omega }$ is the weight matrix, ${MAT}_{FLT}$ is the filter parameters matrix, $\omega_{i}$ is the weight of the *i*-th filter, ${FLT}_{n}^{i}$ is the internal parameters of the *i*-th filter, *n* is the number of parameters in each filter, Comb_FLT is the parameters of the Combined Filter, ⊕ is element-wise addition and ${Conv}_{Comb\_FLT}$ is the convolution operation using the Combined Filter.

**(2)**  **BiLSTM module (Decoder)**

The BiLSTM module uses a BiLSTM network to learn bidirectional global features and outputs the reconstruction results of protein primary structure through the output layer. The BiLSTM network contains forward and backward connections of LSTM cells, and each LSTM cell has three gating mechanisms: an input gate, forget gate and output gate. The calculation process of a sequence of length *L* in an LSTM cell is as follows:

(8) $$f_{t}=\sigma \left(W_{f}\left[h_{t-1},x_{t}\right]+b_{f}\right)$$

(9) $$i_{t}=\sigma \left(W_{i}\left[h_{t-1},x_{t}\right]+b_{i}\right)$$

(10) $$o_{t}=\sigma \left(W_{o}\left[h_{t-1},x_{t}\right]+b_{o}\right)$$

(11) $$C_{t}=f_{t}⊙C_{t-1}+i_{t}⊙\tanh\left(W_{C}\left[h_{t-1},x_{t}\right]+b_{C}\right)$$

(12) $$h_{t}=o_{t}⊙\tanh\left(C_{t}\right)$$

where σ is the sigmoid function, tanh is the hyperbolic tangent function, ⊙ is element-wise multiplication, $t\in \left\{1,2,\cdots ,L\right\}$ and $x_{t}$, $h_{t}$ and $C_{t}$ are the input feature, output feature and cell state at the *t*-th time step, respectively. *W* and *b* are the weight and bias parameters, respectively. $f_{t}$, $i_{t}$ and $o_{t}$ are the outputs of the forget gate, input gate and output gate, respectively.

Since HTA can generate specific Combined Filters for different sequences, it is highly targeted to sample information. Furthermore, each masked segment has a single secondary structure category; HTA is highly targeted to protein secondary structure.

#### 4.3.2. PSSP-MTL Model

The PSSP-MTL model is the main part of MHTAPred-SS, which consists of three modules. First, the concatenated feature of the hybrid feature and the highly targeted encoding feature is input into the multi-scale residual convolution module to extract multi-scale local features. Since embedding features contain information between related residues, it is of little significance for the multi-scale residual convolution module to act on embedding features. Therefore, embedding features are directly concatenated with multi-scale local features and input into the MMoE structure. In particular, we introduce residual connections in each module of the PSSP-MTL model, which can reduce information loss and effectively alleviate gradient vanishing and gradient explosion problems.

**(1)**  **Multi-scale residual convolution module**

As shown in Figure 12, the multi-scale residual convolution module we designed contains multiple single-scale convolutions and residual connections, aiming to extract multi-scale local features based on the knowledge of secondary structure segment length pattern. The embedding features extracted by ESM-2 are concatenated with multi-scale local features to generate the concatenated feature tensor as follows:

(13) $$F_{C}=Concat\left(LeakyReLU\left({{Res}_{1}⊕F}_{1}\right),\cdots ,LeakyReLU\left({{Res}_{s}⊕F}_{s}\right),F_{embed}\right)$$

where *s* is the number of scales, $F_{i}$ is the output features of the *i*-th single-scale convolution, ${Res}_{i}$ is the features transmitted by the *i*-th residual connection, $F_{embed}$ is the embedding features, Concat is the concatenation operation and $F_{C}$ is the concatenated feature tensor.

**(2)**  **Parameter sharing module**

Compared with the STL strategy, MTL can achieve better generalization performance through information exchange between tasks. It is mainly divided into hard parameter sharing models such as MT-DNN [51] and soft parameter sharing models such as MMoE [52]. The former refers to completely sharing bottom parameters; it is suitable for tasks with very strong correlation. The latter refers to partially sharing bottom parameters; it does not require very strong correlation between tasks and can better adapt to requirements of different tasks. Particularly, MMoE is highly suitable for scenarios with fewer processing tasks. Based on the task correlation knowledge and the number of tasks, we adopt the MMoE structure to achieve information exchange between tasks. The task correlation knowledge will be introduced in detail in Section 3.1.2.

As the bottom part of the MMoE structure, the parameter sharing module we designed consists of three parts: residual connections, gating networks and expert networks. The numbers of residual connections and gating networks are the same as the number of tasks, and the number of expert networks is a hyperparameter that can be tuned. We assume that the number of tasks is $N_{T}$ and the number of expert networks is *E*.

The input of the parameter sharing module is concatenated feature tensor of multi-scale local features and embedding features. The parameter sharing module comprehensively considers the output features of all expert networks and finally generates fusion features.

Each residual connection utilizes a Conv1D and a BN to process the concatenated feature as follows:

(14) $$F_{Res}^{j}=BN\left(Conv\left(F_{C}\right)\right)$$

where $j\in \left\{1,2,\cdots N_{T}\right\}$ and $F_{Res}^{j}$ is the features transmitted by the *j*-th residual connection.

Each gating network consists of Conv1D, BN, LeakyReLU function and Softmax operation, and the output is the weights assigned to expert networks as follows:

(15) $$F_{G}^{j}=Softmax\left(LeakyReLU\left(BN\left(Conv\left(F_{C}\right)\right)\right)\right)$$

(16) $$F_{G}^{j}=\left[\begin{array}{c} \omega_{j}^{1}\\ \omega_{j}^{2}\\ \vdots \\ \omega_{j}^{E} \end{array}\right]={\left[\begin{array}{ccccc} \omega_{j}^{1\left(1\right)} & \omega_{j}^{1\left(2\right)} & \cdots & \omega_{j}^{1\left(L\right)} & \\ \omega_{j}^{2\left(1\right)} & \omega_{j}^{2\left(2\right)} & \cdots & \omega_{j}^{2\left(L\right)} & \\ \vdots & \vdots & \ddots & \vdots & \\ \omega_{j}^{E\left(1\right)} & \omega_{j}^{E\left(2\right)} & \cdots & \omega_{j}^{E\left(L\right)} \end{array}\right]}_{E\times L}$$

where $F_{G}^{j}$ is the output feature tensor of the *j*-th gating network, $\omega_{j}^{k}$ is the weights assigned by the *j*-th gating network to the output feature of the *k*-th expert network and *L* is the length of the sequence.

**Figure 14:**
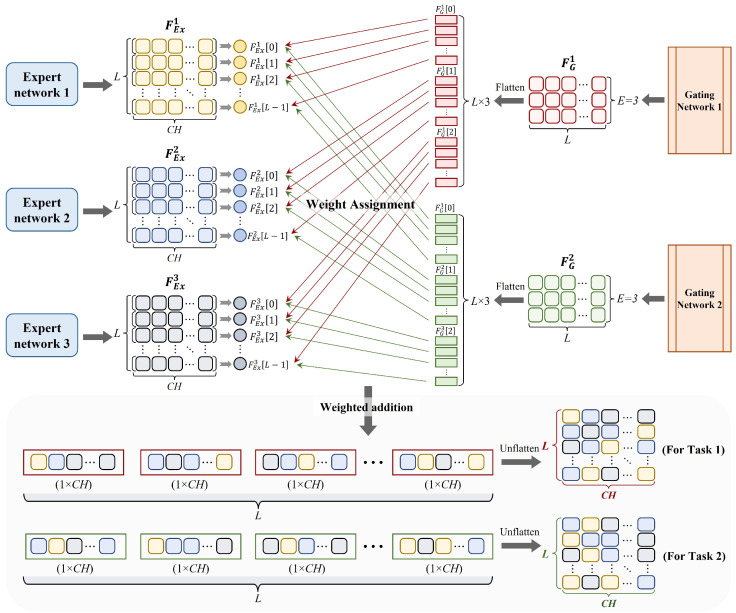
The weighted fusion principle of features output by expert networks. “Weight Assignment” aims to assign weights to the output features of each expert network, and “Weighted addition” means aggregating the output features of each expert network according to the assigned weights to obtain the features extracted for each task.

Each expert network consists of β Conv1D and BN combinations. The output features of expert networks are weightedly added to obtain the fusion feature for each task. The weighted fusion principle is shown in Figure 14 and the calculation process is as follows:

(17) $$F_{EX}^{k}={EX}_{k}\left(F_{C}\right)$$

(18) $$F_{Output}^{j}=LeakyReLU\left(F_{Res}^{j}⊕\sum_{k=1}^{E}F_{Ex}^{k}⊙{\left[\begin{array}{c} \omega_{j}^{k}\\ \vdots \\ \omega_{j}^{k} \end{array}\right]}_{CH\times L}\right)$$

where $k\in \left\{1,2,\cdots E\right\}$, ${EX}_{k}$ is the operation inside the *k*-th expert network, $F_{EX}^{k}$ is the output feature tensor of the *k*-th expert network, $F_{Output}^{j}$ is the fusion feature tensor extracted by the parameter sharing module for the *j*-th task and CH is the channel number of $F_{Output}^{j}$.

**(3)**  **Combined time series module**

**Figure 15:**
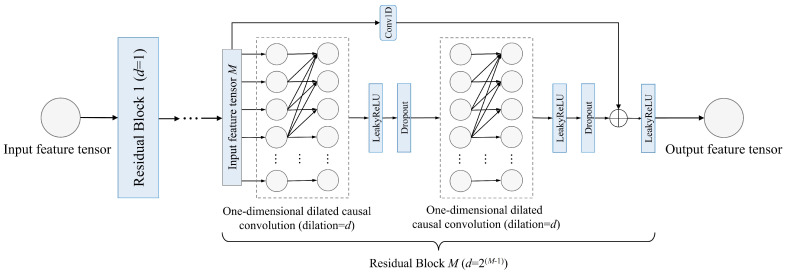
The architecture of the TCN unit.

We built the combined time series module as the tower part of the MMoE structure, which cascades a temporal convolutional network (TCN) [53] and bidirectional gated recurrent unit (BiGRU) [31]. The architecture of the TCN unit is shown in Figure 15, which consists of *M* residual blocks. The *i*-th residual block contains two one-dimensional dilated causal convolution layers with a dilation rate of $2^{\left(i-1\right)}$.

The BiGRU unit uses a BiGRU network to extract global features. Similar to the BiLSTM network, the BiGRU network contains forward and backward connections of gated recurrent unit (GRU) cells, but it has fewer parameters and requires less computing resources and memory. The GRU cell contains two gating mechanisms, namely reset gate and update gate, which are defined as follows:

(19) $$r_{t}=\sigma \left(W_{r}\left[h_{t-1},x_{t}\right]+b_{r}\right)$$

(20) $$z_{t}=\sigma \left(W_{z}\left[h_{t-1},x_{t}\right]+b_{z}\right)$$

(21) $$h_{t}=z_{t}⊙h_{t-1}+\left(1-z_{t}\right)⊙\tanh\left(W_{h}\left[{r_{t}⊙h}_{t-1},x_{t}\right]+b_{h}\right)$$

where $t\in \left\{1,2,\cdots ,L\right\}$. $x_{t}$ and $h_{t}$ are the input feature and output feature at the *t*-th time step, respectively. *W* and *b* are the weight and bias parameters, respectively. $r_{t}$ and $z_{t}$ are the output of reset gate and update gate, respectively.

The TCN unit can process sequence data in parallel, significantly improving data processing efficiency, and residual connections can enhance the robustness of the model. The BiGRU unit can enhance learning capabilities of the model, and supplement the unidirectional features extracted by the TCN unit. In summary, the combination of two time series models with different advantages can effectively avoid limitations of single models to fully extract global features of sequences.

### 4.4. Evaluation Metrics

We evaluated the prediction performance of models using accuracy metrics calculated for individual residues (Q3 and Q8) and SOV metrics calculated for residue segments (SOV3 and SOV8) [54]. The accuracy value calculated for individual residues is defined as follows:

(22) $$Q\left|SS\right|=\frac{\sum_{i\in SS}T_{i}}{sum}\times 100\%$$

where SS is the set of secondary structure categories, $\left|SS\right|$ is the number of elements in SS (3 or 8), $T_{i}$ is the number of residues with secondary structure category *i* that are correctly predicted and sum is the total number of residues.

The SOV metrics were calculated for residue segments. For the subsequent tertiary structure prediction task, it is more critical to correctly predict secondary structure segments than to correctly predict the secondary structure categories of individual residues. The calculation process of SOV values is as follows:

(23) $$N=\sum_{i\in SS}\left(\sum_{\left(s1,s2\right)\in s\left(i\right)}l\left(s1\right)+\sum_{s1\in \tilde{s}\left(i\right)}l\left(s1\right)\right)$$

(24) $$\delta \left(s1,s2\right)=\min\left\{maxov\left(s1,s2\right)-minov\left(s1,s2\right),minov\left(s1,s2\right),\left[\frac{l(s1)}{2}\right],\left[\frac{l(s2)}{2}\right]\right\}$$

(25) $$SOV\left|SS\right|=\left[\frac{1}{N}\times \sum_{i\in SS}\sum_{\left(s1,s2\right)\in s\left(i\right)}\frac{minov\left(s1,s2\right)+\delta \left(s1,s2\right)}{maxov\left(s1,s2\right)}\times len\left(s1\right)\right]\times 100\%$$

where $s\left(i\right)$ is the set of segment pairs with overlapping experimental and predicted results for category *i*, $\tilde{s}\left(i\right)$ is the set of segments in experimental results for category *i* that does not overlap with predicted results, l(s) is the length of *s*, $minov\left(s1,s2\right)$ is the number of residues in the secondary structures of s1 and s2 that overlap and $maxov\left(s1,s2\right)$ is the total number of residue positions in s1 and s2.

### 4.5. Experimental Details

We used an NVIDIA Corporation Device 2684 GPU and 12th Gen Intel(R) Core(TM) i7-12700F CPU to perform experiments based on the PyTorch deep learning framework. The cross-entropy loss function and the AdamW optimizer with a weight decay of 0.05 were used for experiments. The batch size was set to 32, and all sequences were padded to the length of the longest sequence in the batch. We set the initial learning rate to 0.0005, and reduced it to half of the previous value every five training epochs. The specific model hyperparameter setting of MHTAPred-SS is provided in Supplementary Table S2.

## 5. Conclusions

In this study, we proposed MHTAPred-SS, a novel PSSP framework. In the proposed MHTAPred-SS, we designed HTA as a driver, which can extract highly targeted encoding features through the attention mechanism on the sample dimension and the random secondary structure segment masking. HTA does not require a time-consuming MSA process, and can improve feature space utilization of PSSP-related knowledge. Moreover, we designed the PSSP-MTL model based on biological knowledge as the main part of MHTAPred-SS. The PSSP-MTL model balances global and multi-scale local features based on multi-scale residual convolution and combined time series networks, and adopts an MMoE structure for information exchange between tasks. Through extensive ablation experiments, we demonstrated that the short and homologous protein-independent highly targeted encoding features can play a comparable role with profile features, and the MMoE structure enables the auxiliary task to effectively assist the PSSP task. In addition, we compared MHTAPred-SS with 10 baselines on six independent test sets. It can be seen from experimental results that MHTAPred-SS achieves state-of-the-art performance and has significant advantages in single-category and boundary secondary structure prediction. It can also be intuitively observed from the case study that MHTAPred-SS can flexibly and accurately predict the distribution of secondary structure segments, which also confirms the fact that MHTAPred-SS has remarkable performance in SOV metrics.

Currently, based on advanced protein molecular sequencing technology, the scale between protein sequence data and structural data is growing exponentially, and advanced protein overall structure prediction tools have high requirements for storage and computing resources and cannot be flexibly used locally. The MHTAPred-SS we proposed can achieve relatively accurate protein secondary structure prediction under limited resource conditions, thereby facilitating the determination of the overall protein structure and thus expanding the scale of the protein structure database. In future work, we will also use the predicted results as biological features for subsequent tasks such as protein–protein interaction and drug–target affinity prediction, which is expected to achieve new breakthroughs in areas such as protein engineering and drug discovery. 

## Figures and Tables

**Table 1:** The contingency table of secondary structure and RSA.

	SS	H	G	I	E	B	T	S	L	Sum
RSA										
≤0.15		1,000,536	82,456	643	813,132	29,114	198,608	180,445	479,341	2,784,275
>0.15		999,976	141,550	391	466,328	32,103	446,346	293,307	865,191	3,245,192
Sum		2,000,512	224,006	1034	1,279,460	61,217	644,954	473,752	1,344,532	6,029,467

Note: “SS” represents protein secondary structure.

**Table 2:** The number of boundary amino acid residues contained in six independent test sets.

Types	DS1 Test Sets				DS2 Test Sets	
	CASP12	CASP13	CASP14	CB513	TEST2016	TEST2018
#Boundary3	3995	3591	2513	28,306	93,219	17,243
#Boundary8	6139	5588	3874	44,360	142,402	26,103

Note: “#Boundary3” represents the number of 3-state boundary amino acid residues. “#Boundary8” represents the number of 8-state boundary amino acid residues.

**Table 3:** The prediction performance of models when using different numbers of filters in residual DyConv1D blocks.

*B*	Validation Set1		Test Set1		Validation Set2		Test Set2	
	Q3 (%)	Q8 (%)	Q3 (%)	Q8 (%)	Q3 (%)	Q8 (%)	Q3 (%)	Q8 (%)
1	88.16	**78.96**	87.12	**76.55**	88.26	78.96	87.96	**78.62**
2	88.09	78.86	87.16	76.46	88.34	79.08	87.99	78.58
3	**88.18**	78.80	87.17	**76.55**	**88.43**	**79.11**	**88.04**	78.61
4	88.02	78.73	**87.19**	76.27	88.26	79.00	87.88	78.59

Note: the best results are shown in bold.

**Table 4:** The prediction performance of models under different convolution scales.

Scales	Validation Set1		Test Set1		Validation Set2		Test Set2	
	Q3 (%)	Q8 (%)	Q3 (%)	Q8 (%)	Q3 (%)	Q8 (%)	Q3 (%)	Q8 (%)
1	**88.28**	79.03	87.40	76.21	88.45	79.07	88.11	78.50
3	88.19	78.65	87.23	76.42	88.12	79.07	87.82	78.67
5	88.21	78.67	87.32	76.23	88.46	79.06	88.02	78.69
7	88.04	**79.06**	87.31	76.40	88.43	79.10	87.92	78.51
9	88.17	79.04	87.26	76.47	88.39	79.17	87.96	78.55
11	88.17	78.85	87.09	76.32	88.30	78.88	87.95	78.46
(1,5,9)	88.27	79.02	**87.43**	**76.53**	**88.52**	**79.18**	**88.14**	**78.74**

Note: “(1,5,9)” represents multi-scale residual convolution module at three scales: 1, 5 and 9. Underlined data represent the best results at a single scale. The best results are shown in bold.

**Table 5:** The prediction performance of models with different numbers of expert networks.

*E*	Validation Set1		Test Set1		Validation Set2		Test Set2	
	Q3 (%)	Q8 (%)	Q3 (%)	Q8 (%)	Q3 (%)	Q8 (%)	Q3 (%)	Q8 (%)
1	87.88	78.67	87.02	76.00	88.14	78.77	87.70	78.28
2	88.12	78.87	87.14	76.52	88.48	79.03	**88.17**	78.58
3	**88.27**	**79.02**	**87.43**	**76.53**	**88.52**	**79.18**	88.14	**78.74**
4	88.17	78.93	87.32	76.40	88.31	79.04	87.98	78.59

Note: the best results are shown in bold.

**Table 6:** The prediction performance with each component disabled.

Components			Validation Set1		Test Set1		Validation Set2		Test Set2	
#T	#S	#R	Q3 (%)	Q8 (%)	Q3 (%)	Q8 (%)	Q3 (%)	Q8 (%)	Q3 (%)	Q8 (%)
✕	✓	✓	87.99	78.69	87.15	76.08	88.16	78.71	87.81	78.37
✓	✕	✓	87.99	78.70	87.07	76.16	88.29	78.77	87.94	78.38
✓	✓	✕	88.13	78.95	87.38	76.52	88.48	79.12	88.12	78.72
✓	✓	✓	**88.27**	**79.02**	**87.43**	**76.53**	**88.52**	**79.18**	**88.14**	**78.74**

Note: “#T” represents the combined time series module. “#S” represents the parameter sharing module. “#R” represents residual connections in multi-scale residual convolution and parameter sharing modules. “✓” represents using this component. “✕” represents disabling this component. The best results are shown in bold.

**Table 7:** The prediction performance when each component is used individually.

Components			Validation Set1		Test Set1		Validation Set2		Test set2	
#T	#S	*R	Q3 (%)	Q8 (%)	Q3 (%)	Q8 (%)	Q3 (%)	Q8 (%)	Q3 (%)	Q8 (%)
✓	✕	✕	88.00	78.75	87.16	76.22	88.33	78.85	87.89	78.42
✕	✓	✕	87.79	78.51	86.72	75.74	87.99	78.41	87.60	78.10
✕	✕	✓	87.52	77.65	86.35	74.76	87.53	76.29	87.14	77.61
✓	✓	✓	**88.27**	**79.02**	**87.43**	**76.53**	**88.52**	**79.18**	**88.14**	**78.74**

Note: “#T” represents the combined time series module. “#S” represents the parameter sharing module. “*R” represents the multi-scale residual convolution module. “✓” represents using this component. “✕” represents disabling this component. The best results are shown in bold.

**Table 8:** The prediction performance under different feature combinations.

Features					Validation Set1		Test Set1		Validation Set2		Test Set2	
#ESM	#H_E_	#P	#H	#P_S_	Q3 (%)	Q8 (%)	Q3 (%)	Q8 (%)	Q3 (%)	Q8 (%)	Q3 (%)	Q8 (%)
✕	✓	✓	✓	✓	85.30	74.53	84.83	72.60	85.17	74.90	85.06	74.50
✓	✕	✓	✓	✓	88.09	78.92	87.06	76.37	88.13	78.94	87.80	78.64
✓	✓	✕	✓	✓	88.03	78.83	87.08	76.11	88.24	78.81	87.85	78.36
✓	✓	✓	✕	✓	88.11	78.79	86.97	76.03	88.36	78.71	87.91	78.34
✓	✓	✓	✓	✕	88.25	78.77	87.26	76.42	**88.61**	79.13	88.12	78.61
✓	✕	✕	✕	✕	87.72	78.23	86.14	75.24	87.74	78.26	87.23	77.76
✕	✓	✕	✕	✕	71.38	59.80	70.61	56.67	71.63	59.43	71.78	59.32
✕	✕	✓	✕	✕	83.40	72.26	82.93	69.99	83.67	71.88	83.35	71.67
✕	✕	✕	✓	✕	82.77	71.47	83.11	70.00	83.26	72.35	82.89	71.90
✕	✕	✕	✕	✓	72.70	59.97	71.90	56.86	72.32	59.65	72.33	59.78
✓	✓	✓	✓	✓	**88.27**	**79.02**	**87.43**	**76.53**	88.52	**79.18**	**88.14**	**78.74**

Note: “#ESM” represents the embedding feature extracted by the ESM-2 model. “#H_E_” represents the highly targeted encoding feature. “#P” represents the PSSM profile feature. “#H” represents the HMM profile feature. “#P_S_” represents physicochemical properties. “✓” represents using this feature. “✕” represents disabling this feature. The best results are shown in bold.

**Table 9:** The prediction performance comparison on the independent test sets of DS1.

*i*-State	Methods	CASP12		CASP13		CASP14		CB513	
		Q*_i_* (%)	SOV*_i_* (%)	Q*_i_* (%)	SOV*_i_* (%)	Q*_i_* (%)	SOV*_i_* (%)	Q*_i_* (%)	SOV*_i_* (%)
i=3	DCRNN	83.91	71.61	82.30	69.47	79.08	60.05	84.28	79.89
	DeepCNN	84.79	71.98	82.66	63.82	78.86	59.45	84.47	78.84
	CNN_BIGRU	83.84	73.45	83.11	69.30	79.43	63.29	84.52	80.22
	MUFOLD-SS	84.36	73.00	83.43	67.97	78.72	60.05	84.44	79.96
	DeepACLSTM	83.90	72.47	82.93	68.34	78.51	61.00	84.51	80.48
	DML_SS^embed^	86.08	73.46	84.95	71.07	80.75	65.81	86.41	82.39
	**MHTAPred-SS**	**86.73**	**78.54**	**86.04**	**74.04**	**83.21**	**73.05**	**87.43**	**83.61**
i=8	DCRNN	72.95	61.03	70.93	57.06	67.85	53.77	71.34	68.33
	DeepCNN	73.81	60.79	71.92	55.38	67.91	51.78	72.44	69.51
	CNN_BIGRU	73.26	62.31	70.68	57.17	68.17	53.22	71.21	67.65
	MUFOLD-SS	74.32	62.57	71.85	58.54	68.63	52.11	71.63	68.58
	DeepACLSTM	73.93	62.69	71.17	59.55	68.69	54.27	71.84	68.76
	DML_SS^embed^	76.20	62.76	74.17	60.10	70.22	62.23	75.54	73.22
	**MHTAPred-SS**	**77.30**	**65.97**	**76.00**	**62.01**	**72.54**	**66.25**	**76.53**	**74.71**

Note: the best results are shown in bold.

**Table 10:** The prediction performance comparison on the independent test sets of DS2.

*i*-State	Methods	TEST2016		TEST2018	
		Q*_i_* (%)	SOV*_i_* (%)	Q*_i_* (%)	SOV*_i_* (%)
i=3	DCRNN	83.72	78.39	82.75	75.10
	SPIDER3	84.66	75.62	83.84	73.89
	DeepCNN	85.14	79.31	84.16	76.83
	CNN_BIGRU	85.04	81.61	84.17	79.41
	SPOT-1D	86.67	79.52	85.66	78.77
	MUFOLD-SS	85.54	82.31	84.47	79.39
	DeepACLSTM	85.62	82.60	84.66	80.05
	DML_SS^embed^	87.41	84.51	86.82	82.43
	DML_SS^ensemble^	87.66	84.85	86.98	82.57
	Multi-S3P	87.57	79.84	86.46	79.48
	LIFT_SS	87.84	84.76	87.13	82.32
	**MHTAPred-SS**	**88.14**	**84.89**	**87.42**	**82.81**
i=8	DCRNN	72.19	68.63	70.60	65.82
	DeepCNN	74.54	71.56	72.75	69.18
	CNN_BIGRU	73.91	70.92	72.78	68.75
	SPOT-1D	76.03	73.88	74.26	71.45
	MUFOLD-SS	75.42	73.27	73.66	70.56
	DeepACLSTM	75.19	73.67	73.42	71.32
	DML_SS^embed^	78.03	75.90	76.48	73.44
	DML_SS^ensemble^	78.41	76.44	76.79	74.14
	Multi-S3P	77.56	75.29	76.12	73.36
	LIFT_SS	78.70	76.79	76.86	74.24
	**MHTAPred-SS**	**78.74**	**77.15**	**77.28**	**74.79**

Note: the experimental of LIFT_SS is based on the optimal fine-tuning model. The best results are shown in bold.

**Table 11:** Boundary prediction and overall prediction accuracy on TEST2016.

Methods	Accuracy of Boundary Prediction (%)		Accuracy of Overall Prediction (%)	
	3-State	8-State	3-State	8-State
MUFOLD-SS	73.10	61.87	85.54	75.42
DML_SS^embed^	76.38	65.39	87.41	78.03
**MHTAPred-SS**	**77.21**	**66.43**	**88.14**	**78.74**

Note: the best results are shown in bold.

**Table 12:** Comparison of prediction time with LocalColabFold.

Methods	Time·50 (s)	Time·100 (s)	Time·150 (s)	Time·200 (s)	Rate (%)
LocalColabFold	4.11692	5.27515	6.77159	7.52676	22.551
MHTAPred-SS (3)	0.02827	0.03165	0.03524	0.03854	10.888
MHTAPred-SS (8)	0.02848	0.03235	0.03473	0.03805	10.168

Note: “MHTAPred-SS (3)” represents the 3-state prediction model of MHTAPred-SS. “MHTAPred-SS (8)” represents the 8-state prediction model of MHTAPred-SS. “Time·N” represents the average prediction time for a sequence of length N. “Rate” represents the average growth rate of prediction time.

**Table 13:** The detailed description of six independent test sets.

Types	DS1 Test Sets				DS2 Test Sets	
	CASP12	CASP13	CASP14	CB513	TEST2016	TEST2018
#Sequences	47	41	33	513	1213	250
#Residues	13,718	12,217	9049	84,119	287,877	56,654

Note: “#Sequences” represents the number of sequences. “#Residues” represents the number of residues.

## Data Availability

The source code and data are available for research and non-commercial use at https://github.com/Runqiu-Feng/MHTAPred-SS (accessed on 12 December 2024).

## Supplementary Materials

The following supporting information can be downloaded at: https://www.mdpi.com/article/10.3390/ijms1010000/s1.
