# Supplementary material for: MHTAPred-SS: A Highly Targeted Autoencoder-Driven Deep Multi-Task Learning Framework for Accurate Protein Secondary Structure Prediction
Source: Int J Mol Sci. 2024 Dec 15;25(24):13444. doi: 10.3390/ijms252413444 (PMC11677681; doi:10.3390/ijms252413444)
Supplement: Supplementary file 1 [file ijms-25-13444-s001.zip › ijms-3337557-supplementary.pdf]

Table S1: Interpretation of Cramer's V[1] .

Values	Interpretation
> 0.25	Very strong
> 0.15	Strong
> 0.10	Moderate
> 0.05	Weak
> 0	No or very weak

Table S2: The specific model hyperparameter setting of MHTAPred-SS.

Models	Components		#Layers	#Kernels	#Channels	#Hidden units
HTA	DY-CNN module		2	[1, 1, 1] (×2)<br>[3, 1, 1] (×4) (×2)	{8, 4}	-
	BiLSTM module	BiLSTM network	2	-	-	{20, 20}
		Output layer	2	-	-	{100, 21}
PSSP-MTL	Multi-scale residual convolution module	Multi-scale convolutions	4 (×3)	[1, 1, 0] (×4)<br>[5, 1, 2] (×4)<br>[9, 1, 4] (×4)	{90, 110, 150, 200} (×3)	-
		Residual connections	1 (×3)	[1, 1, 0] (×3)	{200} (×3)	-
	Parameter sharing module	Gating networks	1 (×2)	[3, 1, 1] (×2)	{3} (×2)	-
		Expert networks	2 (×3)	[3, 1, 1] (×3) (×2)	{1024, 512} (×3)	-
		Residual connections	1 (×2)	[1, 1, 0] (×2)	{512} (×2)	-
	Combined time series module	TCN unit 1	2	[5, 1, 2] (×2) (×2)	{256, 256}	-
		BiGRU unit 1	5	-	-	180
		TCN unit 2	2	[5, 1, 2] (×2) (×2)	{32, 32}	-
		BiGRU unit 2	3	-	-	16
	Output predictor	Predictor 1	3	-	-	{180, 180, 3/8}
		Predictor 2	3	-	-	{8, 8, 2}

Note: "#Layers" represents the number of network layers. "#Kernels" represents the number, scale, stride and padding of convolution kernels. "#Channels" represents the number of convolution channels. "#Hidden units" represents the number of hidden units.

References

[1] Haldun, A. User's guide to correlation coefficients. Turkish journal of emergency medicine 2018, 18, 91.
